# Serum Proteome and Cytokine Analysis in a Longitudinal Cohort of Adults with Primary Dengue Infection Reveals Predictive Markers of DHF

**DOI:** 10.1371/journal.pntd.0001887

**Published:** 2012-11-29

**Authors:** Yadunanda Kumar, Cui Liang, Zheng Bo, Jagath C. Rajapakse, Eng Eong Ooi, Steven R. Tannenbaum

**Affiliations:** 1 Interdisciplinary Research Group in Infectious diseases, Singapore-MIT Alliance for Research and Technology (SMART), Singapore, Singapore; 2 Department of Biological Engineering, Massachusetts Institute of Technology, Cambridge, Massachusetts, United States of America; 3 School of Computer Engineering, Nanyang Technological University (NTU), Singapore, Singapore; 4 DUKE-NUS Graduate Medical School, Singapore, Singapore; University of Rhode Island, United States of America

## Abstract

**Background:**

Infections caused by dengue virus are a major cause of morbidity and mortality in tropical and subtropical regions of the world. Factors that control transition from mild forms of disease such as dengue fever (DF) to more life-threatening forms such as dengue hemorrhagic fever (DHF) are poorly understood. Consequently, there are no reliable methods currently available for early triage of DHF patients resulting in significant over-hospitalization.

**Methodology/Principal Findings:**

We have systematically examined the proteome, cytokines and inflammatory markers in sera from 62 adult dengue patients (44 DF; 18 DHF) with primary DENV infection, at three different times of infection representing the early febrile, defervescence and convalescent stages. Using fluorescent bioplex assays, we measured 27 cytokines in these serum samples. Additionally, we used multiple mass spectrometry methods for iTRAQ-based comparative analysis of serum proteome as well as measurements of protein adducts- 3-nitrotyrosine and 3-chlorotyrosine as surrogate measures of free radical activity. Using multiple methods such as OPLS, MRMR and MSVM-RFE for multivariate feature selection and classification, we report molecular markers that allow prediction of primary DHF with sensitivity and specificity of >80%.

**Conclusions/Significance:**

This report constitutes a comprehensive analysis of molecular signatures of dengue disease progression and will help unravel mechanisms of dengue disease progression. Our analysis resulted in the identification of markers that may be useful for early prediction of DHF during the febrile phase. The combination of highly sensitive analytical methods and novel statistical approaches described here forms a robust platform for biomarker discovery.

## Author Summary

While the majority of patients who exhibit febrile dengue infection recover within a week, a small proportion of the patients progress to develop severe symptoms that can be life-threatening if not managed in a hospital setting. Because there is no method to accurately identify this subgroup of patients, many dengue patients are hospitalized unnecessarily, which causes significant burden to the healthcare system. In our study, we have systematically measured a large number of molecules including cytokines and serum proteins in blood samples from a dengue patient cohort using highly sensitive mass spectrometry-based methods. We have further developed novel statistical methods that allow us to identify small panels of measureable blood markers, which can distinguish dengue patients that develop milder, self-limiting form of the disease from those that progress to develop severe symptoms. Because these markers can be applied within 48–72 hours of onset of febrile symptoms, we expect them to be useful for early classification of severe dengue disease.

## Introduction

Infection with dengue virus (DENV) causes a spectrum of clinical manifestations ranging from mild dengue fever (DF) to the potentially lethal dengue hemorrhagic fever (DHF) and dengue shock syndrome (DSS) [1]. In humans, the major cellular targets of dengue appear to be dendritic cells of the skin, macrophages and monocytes [2]. Dengue is endemic to the tropical and sub-tropical regions of the world, which are home to over half the population of the world as well as being popular tourist destinations. It has also emerged in new areas such as south Florida and Mediterranean France. With a significant proportion of the world population at risk of infection annually, coupled with the absence of a licensed vaccine, dengue is emerging as a global health concern.

The majority of dengue patients recover uneventfully after 5–7 days of acute illness. In a small proportion of patients, however, the initial febrile period is followed by a rapid onset of vascular leakage, thrombocytopenia and hemorrhage indicating DHF. The continual loss of intravascular volume from plasma leakage can very rapidly lead to hypotension and cardiovascular collapse which, if not carefully managed, can result in death. In the absence of an effective antiviral drug, the management of dengue patients is primarily supportive. Early recognition of patients with plasma leakage is thus critical for the initiation of appropriate fluid management to prevent onset of hypovolemic shock. However, because these symptoms become evident only in the critical phase of infection, it is currently not possible to distinguish DF and DHF accurately during the early stages of illness, when the disease is less well differentiated [3].

The mechanisms that trigger transition from mild DF to more life threatening DHF are poorly understood, hampering early classification of dengue patients who will progress to DHF. This not only delays treatment but frequently results in the over-hospitalization of patients contributing significantly to the financial burden imposed by dengue [4], [5]. The availability of reliable markers that predict DHF during the early stages of infection could be useful in triaging patients for management.

In the last decade, numerous efforts have been made to identify serum markers that may predict severe dengue disease, with an emphasis on cytokines [6]. A number of studies propose that innate immune cytokines (e.g. IFN-α, IL-8) are elevated during the early febrile phase while adaptive immune cytokines (e.g. TNF-α, IL-10, IFN-γ) appear to increase during the defervescence phase of dengue infection [6]. Several factors have traditionally limited the usefulness of these studies in biomarker development. Firstly, the highly variable nature of patient cohorts (e.g. pediatric versus adults; ethnicity) used makes it difficult to compare the results of these various studies. Secondly, most studies have examined ‘case versus control’ type of sample population instead of longitudinal studies to distinguish ‘predictors’ from ‘indicators’. Finally, a lack of follow-up in larger population base to test the prognostic potential of proposed markers limits their clinical application.

The early dengue infection and outcome (EDEN) study in Singapore prospectively recruits and follows-up adult dengue patients in Singapore through early febrile, defervescence as well as convalescence stages [7] of the disease. This makes this longitudinal study highly suited for the identification of prognostic markers of severe dengue disease. In this study, we report a systematic characterization of serum cytokines, proteome, and markers of macrophage and neutrophil activity in a subset of adult dengue patients with primary dengue infections obtained from the EDEN cohort. In addition to identifying molecular signatures of disease progression, we describe a comprehensive multivariate statistical analysis to identify serum markers for early prediction of DHF.

## Methods

### Dengue patient recruitment, sample collection and clinical evaluation

The EDEN study is a multi-center longitudinal study of adult febrile infections that was carried out at a number of clinics island-wide in Singapore. Enrollment of eligible individuals was based on written informed consents and the protocols were approved by the National Healthcare Group (DSRB B/05/013). The study protocols have been described earlier [7]. In brief, adult patients (>21 years) presenting with acute onset fever (≥38.0°C for less than 72 hours) without rhinitis or other clinical alternatives were included in the study. Initial dengue diagnosis and viremia levels were determined by real time RT-PCR using a previously described method [8]. This was followed by serology and subsequent serotyping by virus isolation and immunofluorescence using serotype specific monoclonal antibodies (ATCC: HB46-49). Venous blood samples were also collected at fever day 4 to 7 (visit-2) and weeks 3 to 4 (visit-3), aliquoted and frozen at −80°C. ‘Fever day’ here refers to number of days post onset of fever. Classification of DF or DHF was made based on the guidelines provided by the WHO [9]. In brief, acute febrile patients positive for dengue with one or more of the following: headache, retro orbital pain, myalgia, rash, leucopenia, hemorrhage were classified as DF while patients with fever lasting 2–7 days combined with bleeding, thrombocytopenia (<100,000/mm^3^) as well as evidence of plasma leakage shown by a 20% or greater rise in hematocrit relative to the blood sample obtained at convalescence or pleural effusion on chest X-ray were classified as DHF. Of the 133 dengue patients that were finally enrolled in this study (September 2005–October 2006), 62 patients (44 DF, 18 DHF) tested negative for dengue IgG antibodies in the acute sera, using a commercial ELISA kit (PanBio, Brisbane, Australia). These patients were deemed to have primary DENV infection, all of which were included in this study.

A detailed hematological and virological analysis was performed and a subset of 15 clinical indicators was selected for our statistical analysis. These included white blood cell count (WBC), red blood cell count (RBC), blood hemoglobin (HGB), hematocrit (HCT), macrophage cell volume (MCV), MCH, MCHC, platelet count (PLT), lymphocyte percentage (LYMPH%), lymphocyte count (LYMPH), mixed cell distribution (MXD), neutrophil percentage (NEUT%), neutrophil count (NEUT), red blood cell distribution width-coefficient of variation (RDW-CV), and viral titers. Additionally, we used plasma samples from 50 asymptomatic healthy army recruits collected during their annual physical examination in Singapore as controls in our analyses. A comparison of cytokines in dengue patient sera with healthy plasma is shown in supplementary data (Figure S1 and Table S1). This study was approved by the National University of Singapore Institutional Review Board and samples were collected with individual informed written consents (see checklist S1).

### Fluorescent bead based measurement of cytokines and serum proteins in patient sera

Cytokine measurements were performed with 12.5 µl sera in duplicates using the Bioplex 27-plex human cytokine kit from BioRad as per manufacturer's instructions. The standard curves were optimized automatically by the software (Bioplex manager) and verified manually. The Bioplex manager software was used to calculate cytokine concentrations and only measurements that showed a coefficient of variability (CV) of <10% were included for further analysis. Levels of interferon-induced cytokine IP-10 in 30% of the dengue patient samples during visit-1 were above upper limit of detection. We repeated the analysis after diluting the serum 100 fold for this subset of samples. Six of the visit-1 samples in DF group and 3 in DHF group still had very high levels of IP-10 and for the statistical analyses, we included these as missing values since levels of other cytokines for these samples were within detectable range. Measurement of 9 acute phase proteins was performed using the Bioplex Pro Acute phase multiplex kit (BioRad laboratories) as per manufacturer's instructions. Two different dilutions of sera were used-1∶1000 for ferritin (FT), serum amyloid A2 (SAA), procalcitonin (PCT), tissue plasminogen activator (tPA), fibrinogen (FB) and 1∶100,000 for alpha-2-macroglobulin (A2M), haptoglobin (HPT), C-reactive protein (CRP) and serum amyloid P (SAP).

### Quantitative analysis of serum proteome

#### Sample pooling, iTRAQ labeling and OFFGEL peptide separation

We performed an isobaric tagging for accurate quantitation (iTRAQ) method for multiplexed analysis of serum proteome in dengue patients. For this, we first pooled serum samples (5 ul each) from 10 DF and 10 DHF patients selected randomly from our study population. The same groups of patients were followed for pooling of the samples from each visit resulting in a total of 6 pools (3 from DF and 3 from DHF). From 10 microliters of each pooled sample, albumin and immunoglobulins were depleted using the Vivapure anti HSA/IgG kit (Sartorius-stedim, USA). Following concentration and desalting, the protein concentration was measured in each sample using the BCA method in a kit [10] (Pierce chemical co. USA). Seventy-five micrograms of protein was subjected with trypsin digestion at a ratio of 1∶80 (trypsin ∶ protein) followed by labeling with isobaric tags using the 4-plex-iTRAQ kit (AB Sciex Pte Ltd, USA) as per manufacturer's instructions. The peptides from DF visit-1 pool were labeled with 115 reporter ions while those from visits 2 and 3 were labeled with 116 and 117 reporter ions respectively. Peptides from an independently processed control serum sample (30 day convalescent sample from a randomly selected dengue patient in our cohort) were labeled with 114 reporter ions. A similar strategy was used in a separate 4-plex experiment for DHF samples but using the same reference ions (114) as the DF samples. The individually labeled peptide samples from each group were pooled and multiplexed peptide samples were desalted using a C-18 SPE cartridge (Agilent technologies), resolved by isoelectric focusing on a pH 3–10 strip (GE healthcare) on an OFFGEL fractionator (Agilent technologies). The resolved peptides were collected in 12 fractions, dried and dissolved in 15 µl of 2%ACN/0.1%TFA.

#### LC MS/MS analysis

The samples were analyzed on an Agilent 6520 Accurate-mass QTOF-LC/MS system equipped with a 1200 series HPLC-Chip/MS system. We separated peptides on a HPLC-Chip with 75 um×150 mm analytical column HPLC-Chip and a 160 nL enrichment column. Three injections (1 ul each with approximately 2 ug peptides) of each sample were separated using a 60 min gradient (5% at 0 min 10% 2 min, 50% 42 min 80% 42–50 min, 5% 50–60 min) with Water/0.1%formic acid as aqueous phase and 95%acetonitrile/5%water/0.1%formic acid as organic phase at flow rates of 300 nL/min. Peptides eluting from the LC were injected online into the accurate-mass QTOF and examined in positive ion mode with the following settings for MS mode: 4 spectra/sec, m/z 110–2300, MS/MS mode: 8 spectra/sec, m/z 60–1097 with drying gas flow 5 L/min, 325degC, collision energy slope 3, intercept 2.5, and a capillary voltage of 1950.

#### Data analysis

Spectrum Mill software (Agilent Technologies) was used for protein identification and quantitation of iTRAQ reporter ion intensities. A minimum peptide score of 8 and a protein score of 10 was used to generate protein lists by searching against the Swissprot database. These thresholds were determined by comparing results from searching the Swissprot database and a reversed random database to identify error rates. The peptide and protein score thresholds indicated above ensured a false discovery rate of <5% in protein identification. Only proteins identified with two or more peptides were selected for relative quantification. A global weighted threshold for fold change was determined by comparing the ratio of summed intensities of each reporter ion for all peptides (115/114- 1.05; 116/114- 0.96; 117/114- 1.09) and then the intensities of each peptide ratio were further corrected by this factor. This weighted threshold was essential to make sure the fold changes observed were not simply due to an overall bias towards one or more reporter ion. Finally, fold change for each protein was calculated as a ratio of summed intensities of reporter ions across different peptides per protein.

### Measurement of serum chlorotyrosine and nitrotyrosine

Nitrotyrosine (NT) and chlorotyrosine (CT) in human serum were measured by a liquid chromatography-triplequadrupole MS method. Briefly, 2 mg of serum protein was spiked with 4 pmol internal standards (IS) L-3-chloro-[^13^C_9_, ^15^N]-tyrosine and L-3-nitro-[^13^C_9_, ^15^N]-tyrosine, and digested in the sodium acetate solution 0.1 M (pH 7.4) with 0.4 mg pronase E (freshly treated by the size-exclusive micro bio-spin column). The mixture was incubated at 50°C for overnight (16 hrs.) and filtered by Vivospin500 3KMW centrifuge filter at 15,000 rpm to remove undigested protein. The amino acids were further purified by Agilent 1200 series HPLC system (Waldbronn, Germany) on an Xbridge TM Phenyl column (3.5 µm, 4.6×50 mm, Waters, Milford, MA). The fractions containing nitrotyrosine and chlorotyrosine, together with internal standards, were collected and dried by SpeedVac for subsequent LC/MS/MS analysis. Subsequent mass spectrometry analysis of target compounds involved separation on an Xbridge TM Phenyl column (3.5 µm, 1.0×100 mm, Waters, Milford, MA) online injection into an Agilent 6460 triple quadrupole mass spectrometer. Two microliters of each sample was injected and eluted by isocratic 25% methanol (0.1% formic acid) for 13 min at 15 µL/min. CT along with IS were analyzed by regular multiple reaction monitoring (MRM) as follows: 216/170 (CT) and 226/179 (CT, IS). NT along with IS were measured by modified MS^3^ based in-source fragmentation as follows: 181/117 (NT) and 190/125 (NT-IS) by elevating the potential to 135 V at the ion source. The limits of quantitation achieved were 8.1 and 7.3 nM for CT and NT, respectively.

### Statistical analysis

#### Clustering of time courses and confidence testing

Overall, our dataset (cytokines, serum proteins and protein adduct measurements) had a largely non-gaussian frequency distribution (D'Agostino and Pearson Omnibus normality test, Graphpad prism), and were unbalanced with unequal sample sizes between different groups necessitating non-parametric data normalization and hypothesis testing where indicated. K-means clustering was performed on time courses of measurements using the Unscrambler-X statistical software package (CAMO software, Oslo, Norway). In order to perform *K*-means clustering of cytokine levels over different time points, mean cytokines levels (population means) were first normalized by dividing the values by their mean (mean of three time points) to bring all cytokine values within range. It was empirically found that *K* = 4 gives the best inter-class variances. In a second step, the members across different clusters were compared manually to determine if the fold changes of cytokines between visit 1 and 2, and visit 2 and 3 were statistically significant (*p*<0.05); if not, the clusters were merged. This resulted in three broad clusters as shown in the results section. For comparison of individual cytokines, serum proteins and protein adducts at different time points or between DF and DHF groups, one-way ANOVA with a non-parametric Kruskal-Wallis test was used. Final diagnosis for this test included DF vs. control, DHF vs. control and DF vs. DHF within each time point. A Dunn's multiple comparison post-test comparing all pairs of datasets was used to test the null hypothesis at a significance threshold p value<0.05. A two-way ANOVA test followed by a bonferroni correction was used to determine if increased proportion of DENV serotype-2 in DHF group compared to DF group affected the outcome of hypothesis testing between them for all cytokines and clinical parameters and was not found to be significant (at p<0.05). Correlation analysis was performed using the Pearson's correlation matrix on the Graphpad prism software and where indicated, the correlation coefficient and the significance values are reported. For all the data analysis performed in this study, p<0.05 was considered significant.

#### Selection of predictive markers

We used multiple feature selection approaches to select subsets (that is, the predictive markers) that give the highest predictive accuracy. To rank the ability of clinical and cytokine measurements to classify different types of dengue populations (e.g. DF vs. DHF), three feature selection strategies were used: the orthogonal projection least squares (OPLS), the multiple-support vector machine-recursive feature elimination (MSVM-RFE), and the maximum relevance minimum redundancy (MRMR) criterion. The OPLS finds features that maximize the correlation between input features and class labels (X) which has been used extensively for biological classification, for example microarray data analysis [11]. Multiple-support vector machine-recursive feature elimination (MSVM-RFE) selects relevant input features, based on the weights of SVM classification, and has been used for selection of genes for sample classification from microarray data [12], [13]. The maximum relevance minimum redundancy (MRMR) criterion ranks features based on the maximum relevancy with the target populations while minimizing the redundancy among all the features [14]. The MRMR was implemented using two measures: the mutual information quotient (MRMR-MIQ) and the mutual information difference (MRMR-MID). Furthermore, the samples were uniformly bootstrapped with replacement and the average rank was computed to stabilize the outcome of feature ranking.

We used the radial basis function (RBF) kernel-based support vector machines (SVM) to evaluate prediction accuracy of each selected subset. The original dataset was partitioned into training and testing datasets, randomly for 1000 times. The regularization and scaling parameters of the RBF kernel SVM were estimated using the leave-one-out cross-validation (LOO-CV) on the training datasets by choosing values selected from a grid formed by the values of regularization and scaling parameters. The hyper-parameters were determined by optimizing the weighted sum of errors in different classes. Here, the weights were used to handle the unbalanced sample sizes of different classes: the error in each class was weighted by the percentage number of samples of another class. The features were then selected by minimizing the same weighted error on the test dataset. This type of analysis was performed using OPLS+SVM, MRMR-MID, MRMR-MIQ, or MSVM-RFE after scaling and standardization as preprocessing of data samples. The performances of features selected by different methods were determined by the area under the curve (AUC) of receiver operating characteristics (ROC). An AUC value of >0.85 was used as a threshold for good predictive performance. Thereafter, one and two-sided two-sample *t*-test were performed on the best bootstrapped accuracy and the accuracies produced by the features selected by other methods. The features selected by the top-performing method and other methods that had no significant difference (p-value>0.05) in accuracies were combined into a common feature set as the top-performing subset. The averages for sensitivity, specificity and AUC of the top-performing feature subsets are reported. A MATLAB script was prepared to run all of the above types of analyses and run on the MATLAB software package. A schematic diagram detailing the workflow for this statistical process is shown in Figure S3.

## Results

### Serum cytokine profile and clinical features of primary dengue infection

We selected 62 adult dengue patients from the EDEN cohort, of which 44 were diagnosed as DF, and 18 as DHF. The patients selected in DF and DHF groups had similar age and ethnic distribution (Table 1). Serotyping analysis indicated that DENV serotypes-1 and -3 were the most common, followed by serotype-2, while no serotype-4 was present. Average duration between fever onset and first sample collection was <48 hours, and the average duration between samples were <80 hours (visit 1 & 2) and <21days (visit 2 & 3) respectively (Table 1).

**Table 1:** Characteristics of dengue patient study population.

Patient Groups	Serotype	Age	Race	Blood sampling Time *
DF (n = 44)	D1 (24, 54%)	39±13.02	Chinese (36, 81.8%)	46±20 hours (visit-1)
	D2 (1, 2.3%)		Indian (2, 4.5%)	73±31 hours (Vist-2)
	D3 (19, 43%)		Malay (2, 4.5%)	16±5 days (visit-3)
	D4 (0)		Others (4, 9.0%)	
DHF (n = 18)	D1 (12, 66.6%)	40±14.08	Chinese (14, 77.7%)	42±22 hours (visit-1)
	D2 (3, 16.6%)		Indian (2, 11.1%)	77±32 hours (Vist-2)
	D3 (3, 16.6%)		Malay (1, 5.55%)	20±12 days (visit-3)
	D4 (0)		Others (1, 5.55%)	

* Average±SD time from fever to phlebotomy (visit-1), between visits 1 & 2 (visit-2) and between visits 2 & 3 (visit-3). DF- dengue fever; DHF dengue hemorrhagic fever; D1-dengue serotype-1; D2-dengue serotype-2; D3-dengue serotype-3, D4-dengue serotype-4.

**Figure 1:**
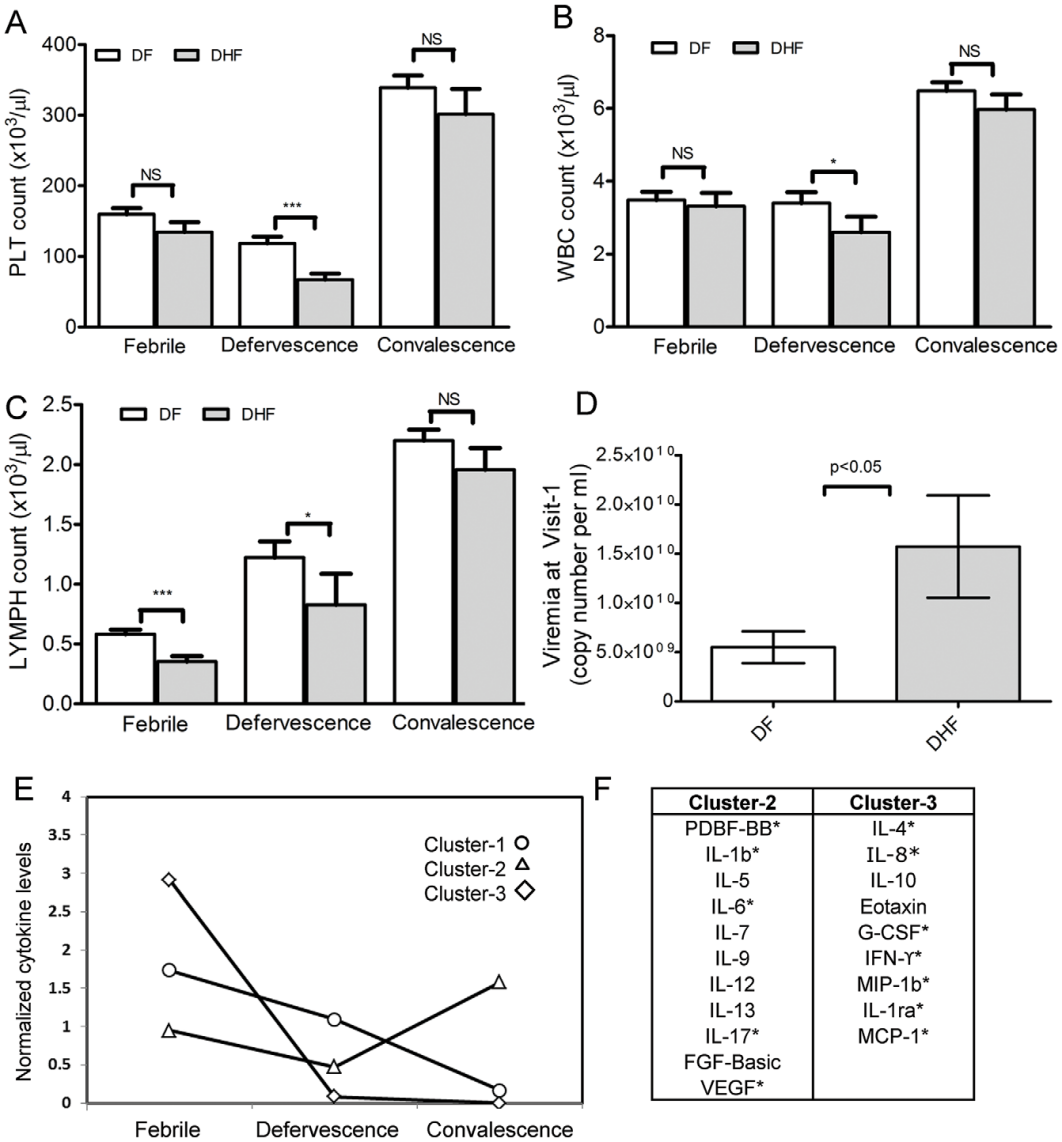
Clinical laboratory features and cytokine responses in primary dengue infections. **A–C** Hematological analysis of patient blood samples (DF (n = 44) and DHF (n = 18)) showing platelet count (A), white blood cell count (B) and lymphocyte count (C). Mean (with upper standard deviation shown in error bars). Statistical confidence was analyzed by ANOVA kruskall-wallis test (DF vs DHF: *p<0.05, **p<0.01, NS- not significant). (D) Viral titers measured by RT-PCR in the febrile stage shown as mean of genomic copies per ml with p values from two-sided student's T-test. E. K-means clustering analysis of temporal profile of 23 cytokines measured at different stages of infection in 62 dengue patients (44 DF+18 DHF). F. Identity of cytokines in each cluster. Cluster-1 consisted of a single cytokine- IP-10, and is not shown in the figure. Cytokines that clustered differently in DF and DHF groups are labeled by asterisk in F.

We examined the key clinical indicators commonly used for the diagnosis of DHF. Blood platelet count dropped significantly from febrile phase to defervescence in both DF and DHF patient groups with DHF patients exhibiting significantly (p<0.05) lower platelet levels during defervescence (visit-2) than DF patients (Figure 1A). DHF patient groups also exhibited significantly (p<0.05) lower WBC and lymphocyte counts especially during defervescence (Figure 1B &C). Viral titer measured at visit-1 was higher in DHF patients (Figure 1D) consistent with several previous studies that have reported higher plasma viral loads in DHF patients [15], [16]. Our study population thus recapitulated most of the hallmark clinical features of dengue progression in DF and DHF during the early febrile, defervescence and convalescence stages of infection.

We measured the levels of 27 serum cytokines in our dengue patient cohort, using a multiplex assay. A majority of cytokines was maximally elevated in dengue patients during the early febrile phase (visit-1) of infection (Table S1, Figure S1). These included IL-1ra, IL-4, IL-7, IL-8, IL-10, IL-12, Eotaxin, G-CSF, IFN-γ, IP-10, MCP-1, MIP-1b and VEGF. Cytokines IL-1b, IL-5, IL-6, IL-9, IL-12, IL-17 and FGF-basic, remained elevated during defervescence (visit-2) and convalescence (visit-3) stages. When compared with plasma samples from an independent cohort of healthy individuals, cytokines IP-10, VEGF and PDGF-BB were found to be elevated >20 times over controls during the febrile phase of infection, while IL-4, IL-9, IL-10 and IL-1ra were elevated by 10–20 times over controls (Table S2). Cytokines IL-6, IL-7, IL-8, Eotaxin, G-CSF, IL-17 and MIP-1b were elevated 4–8 fold (Table S2). These values may however be an overestimation of the actual changes since an independent cohort may not be an ideal control for the study population.

To identify temporal patterns in cytokine flux in patient sera, we performed K-means clustering to group cytokines in DF patients exhibiting similar patterns across the three stages of disease as detailed in the methods section. The cytokine IP-10 was the sole member of cluster-1 (Figure 1E) with very high levels during the febrile phase followed by a rapid decline to near control levels at convalescence. A majority of cytokines fell into a second cluster (Figure 1E and F, Cluster-2) that exhibited a peak at the febrile phase but declined modestly, with levels remaining significantly higher than controls even at the late convalescent stage (visit-3). A third cluster of 7 cytokines (Figure 1E and F, Cluster-3) increased at febrile phase and decreased during defervescence but increased again to peak levels during late stages (visit-3). While overall clustering profile of cytokines was similar between DF and DHF, cytokines IL-1b, IL-4, IL-6, IL-8, IFN-γ, IL-17, G-CSF, VEGF, IP-10, and PDGF-BB (marked by asterisk in Figure 1F) either clustered differently or showed different slopes (not shown) between the disease stages in DF and DHF groups suggesting that there may be changes in the temporal profile of these cytokines.

Overall, our results indicated that cytokines and chemokines associated with innate immune activity (e.g. IFN-γ, IP-10), Th2 cell response (IL-4, IL-10, and IL-13), inflammation (IL-1b, IL-6, and IL-8), chemotaxis of macrophages and neutrophils (Eotaxin, MIP-1b) are all maximally elevated in dengue patients during the early febrile phase. Cytokines IL-12, growth factors FGF and PDGF increased even at convalescence. TNF-α remained below detection levels in our analysis likely because production is transient and missed in our timeline of sample collection. Similarly, levels of IL-2, IL-15, GM-CSF and MIP-1a were below the detection limit in >85% of the samples and were excluded from further analysis.

### Early cytokine responses distinguish DF from DHF

**Figure 2:**
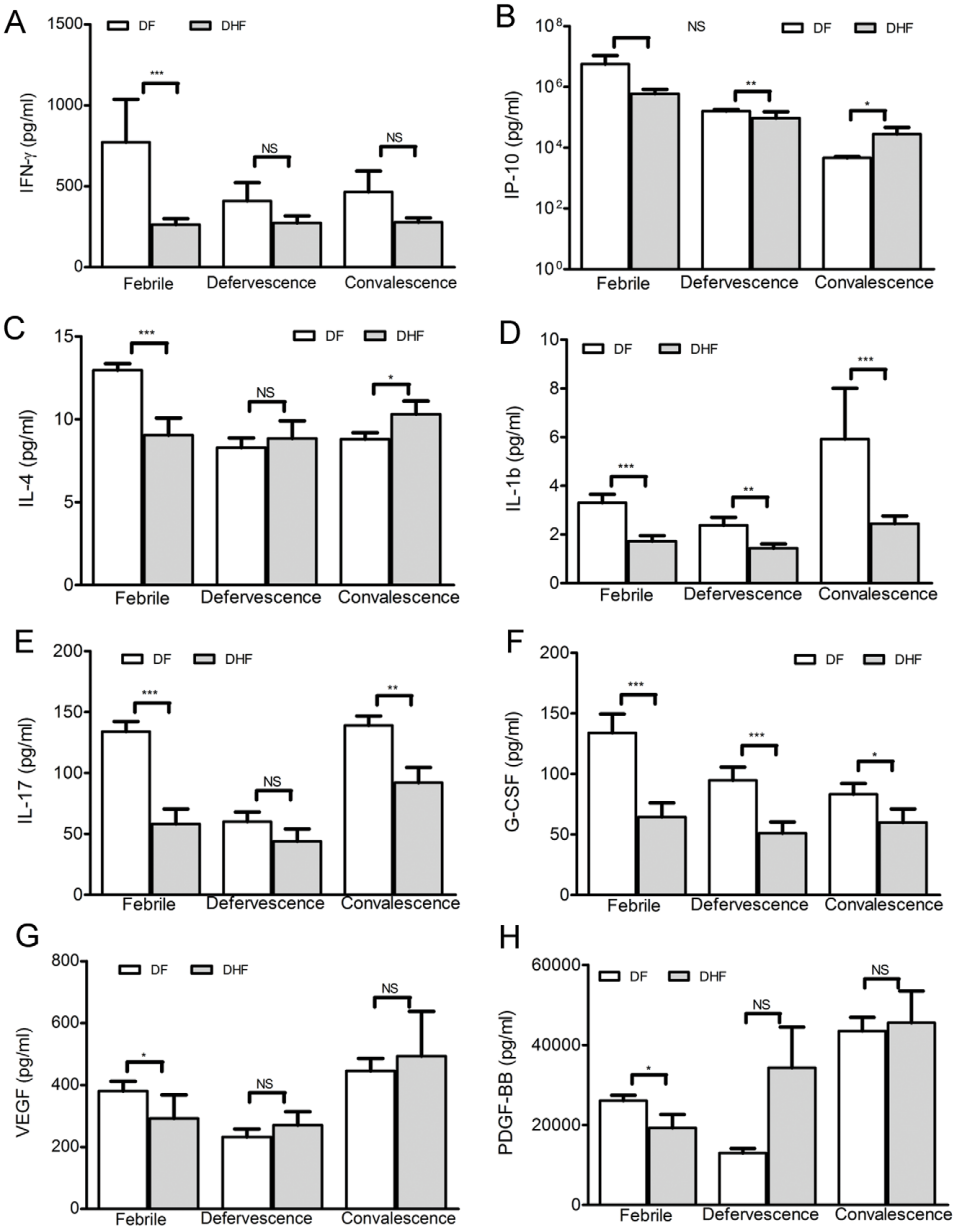
Early cytokine responses distinguish DF and DHF patients. A subset of eight cytokines that exhibited different clustering between DF and DHF patient groups were examined individually. Mean values of IFN-ϒ (A), IP-10 (B), IL-4 (C), IL-1b (D), IL-17 (E), G-CSF (F), VEGF (G) and PDGF-BB (H) from patients (44 DF; 18 DHF) are plotted. Statistical confidence (p<0.05) was analyzed by ANOVA kruskall-wallis test (DF vs DHF: *p<0.05, **p<0.01, ***p<0.001, NS- not significant); dengue (DF or DHF) vs. healthy control: §§§p<0.001, §§p<0.01, §p<0.05, # not significant). Standard deviation from mean across populations is shown in the error bars (upper deviation only).

Differences in temporal profile of a subset of cytokines between DF and DHF patients, identified in the clustering analysis outlined above, prompted us to examine these cytokines more closely across different time points of infection in DF and DHF groups. We observed that DHF patients had lower levels of IFN-γ during febrile phase, a time of peak interferon activity (Figure 2A–B). Although levels of IP-10 (an interferon-induced cytokine) were also lower in the DHF group, this was statistically significant (p<0.05) only at defervescence (Figure 2B). Low levels of IFN-γ as well as IP-10 during the febrile phase point to an attenuated interferon response in DHF patients, which may be associated with diminished viral clearance. There was a marginal but significant correlation between viral titers and IFN-γ levels during the early febrile stage (visit-1, (r = 0.370; p<0.05). The correlation was especially strong between IFN-γ at visit-1 and IP-10 at visit-2 in DHF patients (r = 0.66; p<0.05).

We observed decreased levels of Th2 cytokine IL-4, in DHF patients during the febrile stage, (Figure 2C) compared with DF. Unlike DF patients, IL-1b levels in DHF patients were indistinguishable from healthy controls until the convalescence stage, indicating a depressed IL-1b response (Figure 2D). Levels of IL-17 as well as Granulocyte-Colony Stimulating Factor (G-CSF) were lower in DHF patients especially during the febrile stage (Figure 2E and F). The serum profiles for platelet-derived growth factor (PDGF-BB) as well as vascular endothelial growth factor (VEGF) were similar to G-CSF and markedly lower during the febrile phase in DHF patients compared to DF patients (Figure 2G, H). A similar comparison of other cytokines IL-6 and IL-8 that were found altered in the clustering analysis (Figure 1E &F) indicated that the differences between DF and DHF groups were not statistically significant (data not shown). The number of patients in this study was too low to allow stratification by days from fever onset (Figure S2).

### Profiling of serum proteome flux during dengue progression in humans

**Figure 3:**
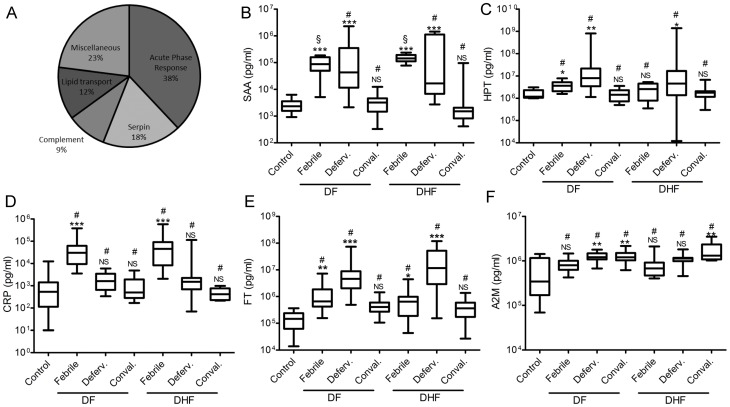
Analysis of serum protein flux at different stages of dengue infections. A. Functional grouping of proteins identified in the proteomics analysis (see Table 2) shown as a pie chart with percent of total proteins (n = 35) identified within each group. B–F. Levels of 9 acute phase reactants measured in sera from 24 DF, 10 DHF patients and 10 healthy control samples using a multiplex assay. Levels of serum amyloid A2 (SAA), haptoglobin (HPT), C-reactive protein (CRP), ferritin (FT) and alpha-2-macroglobulin (A2M) shown in picogram per ml. ANOVA Kruskall-Wallis test- dengue (DHF or DF) vs. Control (*p<0.05, **p<0.01, ***p<0.001, NS- not significant), DHF vs. DF within each time point (§ p<0.05, # not significant). Febrile (visit-1), Deferv. (visit-2) Conval. (visit-3).

Quantitative proteomics by isobaric tagging of peptides allows multiplexing of biological samples thereby reducing variability while increasing accuracy of protein quantitation [17]. We adopted an iTRAQ-based approach to quantify the serum proteome of pooled dengue patient sera during the different stages of the disease. Overall, we identified 90 proteins with high confidence, and determined their fold-change over control samples, in both DF and DHF patient groups (Table 2). Of a total of 35 proteins that showed a >1.5 fold enrichment or depletion, 25 proteins were unique to DHF patient group while 6 proteins - serum amyloid A2, leucine-rich-alpha-2 glycoprotein, hemoglobin alpha, actin, haptoglobin and alpha-1-antitrypsin, changed in both DF and DHF samples (Table 2). The acute phase reactants were the most abundant class, followed by serpin class of protease inhibitors and complement pathway proteins (Figure 3A). A majority of these proteins were maximally elevated during the febrile phase although some remained high or increased further during defervescence (Table 2). Five proteins were depleted from sera during the febrile and defervescence stage but returned to near control levels during the convalescent stage (Table 2). Overall, the proteomic analysis indicated that the most readily observable predominant serum protein response in dengue infections was the acute phase response.

**Table 2:** Serum proteome changes in dengue patient sera across different stages of disease.

Protein	Uniprot ID	Peptides	Functional Class*	Fold Change (DF/DHF)		
				Febrile	Deferv.	Conval.
**Elevated levels in sera during acute infection**						
Serum Amyloid A2**	P02735	3	ACR, HDL	3.4/11.4	1.9/4.7	1.0/1.4
Leucine-rich-alpha-2 glycoprotein**	P02750	8	Unknown	2.0/6.4	1.3/3.6	0.9/2.5
Hemoglobin subunit-alpha**	P69905	4	RBC	1.9/5.9	0.8/1.0	0.8/1.1
Actin, cytoplasmic**	P60709	2	cytoskeleton	2.1/4.2	2.2/7.3	1.2/2.6
Hemoglobin subunit delta	P02042	5	RBC	1.2/4.1	0.9/2.8	0.8/1.3
Insulin-like growth factor-binding protein 3	P17936	7	carrier	1.2/4.0	1.2/3.4	1.0/4.2
Hemoglobin subunit-beta	P68871	5	RBC	1.3/4.0	0.8/2.0	0.7/1.0
Phosphatidyl-inositol glycan specific phospholipase-D	P80108	7	GPI-anchor cleavage	1.2/3.8	0.9/4.0	1.0/2.2
Plasma protease C1 inhibitor	P05155	6	ACR, Comp	1.4/3.8	1.7/5.0	1.0/2.6
Hemoglobin subunit zeta	P02008	11	RBC	1.0/3.5	1.1/1.1	0.9/0.7
Coagulation factor XII	P00748	4	Coagulation	1.3/3.4	1.3/4.2	1.3/3.7
EGF-containing fibulin-like ECM protein	Q12805	4	EGF regulation	1.0/2.9	1.5/2.8	0.9/3.0
Alpha-1B-glycoprotein	P04217	10	Unknown	1.3/2.8	1.2/3.3	1.1/2.8
Tumor protein 63	Q9H3D4	3	DNA-binding	1.5/2.8	0.8/2.5	0.8/3.8
Alpha-2 antiplasmin	P08697	4	ACR, SERPIN	1.2/2.7	1.6/2.5	1.3/2.5
Alpha-1-antichymotrypsin	P01011	18	ACR, SERPIN	1.3/2.5	1.4/2.6	0.8/1.4
Hemopexin	P02790	18	Heme transport	1.0/2.5	0.9/1.8	1.0/1.7
Transthyretin	P02766	4	Hormone binding	0.8/2.4	0.7/0.6	1.3/3.2
Complement C4-A	P0C0L4	34	ACR, Comp	1.3/2.4	1.6/2.2	0.9/1.8
Complement component C9	P02748	4	ACR, Comp	1.5/2.3	1.2/2.9	0.9/1.5
Inter-alpha-trypsin inhibitor H3	Q06033	6	Hyaluronan-bind	0.4/2.3	1.1/2.8	1.3/2.3
Alpha-1-acid glycoprotein 1	P02763	6	ACR	1.5/2.3	1.4/2.2	0.8/1.0
Haptoglobin**	P00738	26	ACR	4.1/2.2	2.0/2.9	2.0/1.5
Antithrombin-III	P01008	17	ACR, SERPIN	1.2/2.2	1.4/3.2	1.1/1.9
Inter-alpha-trypsin inhibitor H2	P19823	19	Hyaluronan-binding	0.8/2.2	0.7/1.9	1.0/2.4
Apolipoprotein A–I	P02647	32	HDL	0.7/2.1	0.9/1.1	0.7/1.5
Serum Paraoxonase/arylesterase 1	P27169	7	HDL	1.0/2.1	1.0/2.1	1.1/1.9
Apolipoprotein E	P02649	15	LDL, HDL	1.3/2.0	1.2/2.8	1.5/2.3
Corticosteroid-binding globulin	P08185	4	SERPIN	0.9/2.0	0.8/1.9	0.9/1.8
Alpha-1-acid glycoprotein 2	P19652	5	ACR	1.4/2.0	1.4/2.7	1.4/1.3
Haptoglobin-related protein	P00739	3	ACR,SERPIN	1.2/2.0	1.5/1.5	0.9/1.0
Clusterin	P10909	12	Chaperone	1.0/2.0	1.0/2.4	1.2/2.2
Alpha-2-HS-glycoprotein	P02765	10	ACR	1.1/2.0	1.3/1.8	0.9/2.4
Alpha-1-antitrypsin**	P01009	20	ACR, SERPIN	1.5/1.9	2.2/3.3	1.1/1.2
**Depleted in sera during acute infection**						
Apoliprotein CI	P02654	2	VLDL	0.3/1.05	0.4/0.9	0.7/1.9
Apoliprotein CII	P02655	3	VLDL	0.4/1.3	0.6/1.6	1.1/2.6
Apoliprotein CIII	P02656	2	VLDL	0.5/0.8	0.6/0.8	1.1/1.2
Apoliprotein CIV	P55056	2	VLDL	0.17/−	0.8/−	0.9/−
Platelet basic protein	P02775	3	Platelet cytokine	0.5/1.2	0.4/0.8	1.0/2.0

* Functional class obtained from Uniprot database (www.uniprot.org); ACR-Acute phase reactant, SERPIN- Serine protease inhibitor, LDL-Low density lipoprotein, HDL-High density lipoprotein, Comp- Complement pathway.

** indicates proteins that changed >1.5 fold in both DF and DHF samples during the febrile or defervescence stage. Fold change – refers to ratio of mass ion (115 –visit-1, 116-visit2, 117-visit-3) intensities over the 114 mass ion intensity representing a reference control sample (see methods) Febrile stage corresponds to visit-1, Deferv. –visit-2, Conval.-visit-3. DF- dengue fever, DHF- dengue hemorrhagic fever.

A major caveat of the sample pooling approach described above is the averaging effect which may result in a gross underestimation of fold changes despite the high accuracy and sensitivity of the proteomic quantification. As an alternative, we used a commercially available multiplex fluorescent-bead based ELISA assay, which simultaneously measures levels of 9 well-known acute phase proteins including two serum proteins (serum amyloid A2 (SAA) and haptoglobin (HPT)) that were identified in our proteomics analysis (Table 2). Using this method, we analyzed individual serum samples from 10 DHF, 24 DF patients and 10 healthy asymptomatic controls. SAA and HPT were elevated in dengue patients during the early febrile (visit-1) and defervescence (visit-2) stages (Figure 3B–C). Other acute phase proteins that were elevated in dengue patients included C-reactive protein (CRP), alpha-2 macroglobulin (A2M) and ferritin (FT) (Fig. 3D–F), while serum amyloid P (SAP), pro-calcitonin (PCT), tissue plasminogen activator (t-PA) and fibrinogen (FB) remained unchanged (not shown). With the exception of SAA, which was higher in DHF patients during the febrile phase, the differences in levels of other proteins between DF and DHF patient groups were not statistically significant.

### Markers of neutrophil and macrophage activity are elevated in dengue patient sera

**Figure 4:**
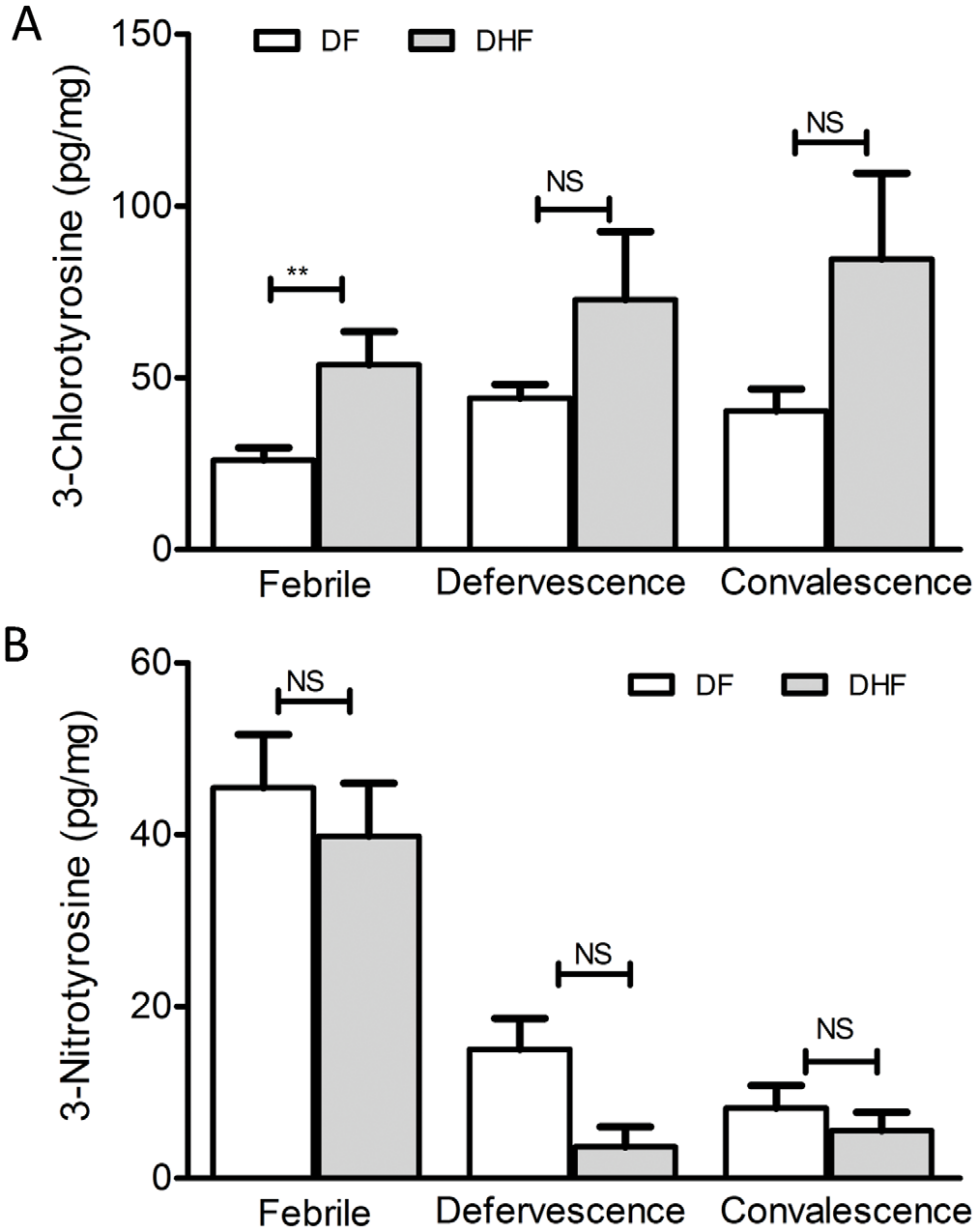
Markers of macrophage and neutrophil activity are in dengue patient sera. Total serum CT (A) and NT (B) levels in dengue patients during febrile, defervescence and convalescence stages. Levels of CT and NT measured in 15 healthy samples was found to be below detection limit (not shown). Statistical confidence was analyzed by ANOVA kruskall wallis-test, DF vs DHF (**p<0.01, NS- not significant).

We used a previously established mass spectrometry based method [18] to measure levels of total serum 3-nitro-tyrosine (NT) and 3-chloro-tyrosine (CT), in 44 DF patients and 10 DHF patients at three different stages of the disease. Compared to healthy individuals where CT and NT levels in sera are below detection, there was a significant elevation of both CT and NT in dengue patient sera (Figure 4). Levels of CT were elevated in all dengue patients during the febrile phase compared to controls, and continued to increase during defervescence and remained high at convalescence (Figure 4A). This suggests that neutrophil activity remains high even after viral clearance. Interestingly, DHF patients displayed higher levels of CT compared to DF patients during the early febrile phase and although higher levels were also seen during defervescence and convalescence, the differences at the latter stages were not statistically significant (at p<0.05). NT peaked during the early febrile phase of the infection but declined to near basal levels during the convalescence stage (Figure 4B). We did not observe statistically significant (p<0.05) differences in NT levels between DF and DHF groups in our experiment.

### Multivariate analysis reveals predictive markers of DHF

**Figure 5:**
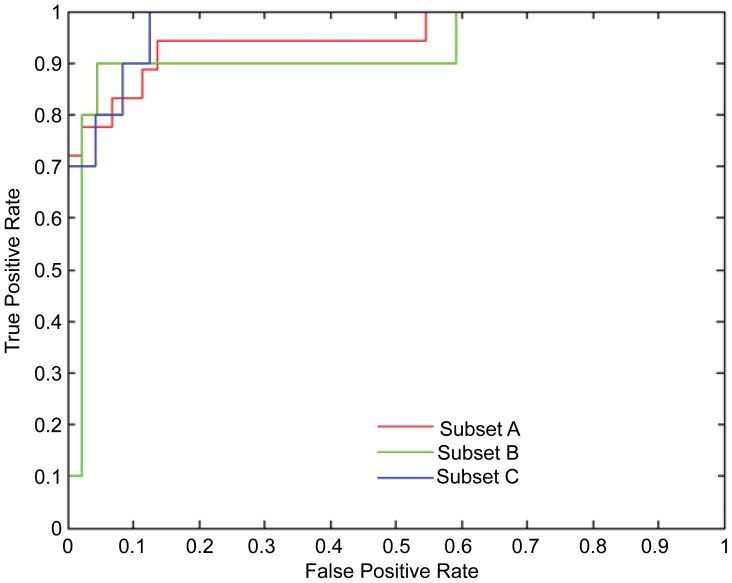
Multivariate statistics reveals predictive serum markers for early classification of DHF. A. Receiver operator characteristic curve (ROC) analysis of subset groups A (cytokines only), B (cytokines+clinical indicators) and C (cytokines+clinical indicators+protein adducts) (see table-3 for subsets) is shown. Area under curve (AUC), values of >0.85, indicated good performance (see methods) with high sensitivity and specificity.

We adopted a multiple-feature selection strategy to identify subsets of features from among the 47 blood parameters described above that may have predictive value in the identification of DHF during the early febrile phase. By analyzing the various feature classes (i.e. cytokines, serum proteins, protein adducts, and clinical features) measured at the early febrile phase (visit-1), both independently, as well as together we evaluated the relative predictive power of these various molecules. First, we analyzed 23 cytokines and identified a subset of 7 cytokines which displayed sensitivities and specificities >75% (Table 3). A receiver operator characteristics (ROC) curve analysis indicated that this subset performed well with area under curve (AUC) of 0.87±0.05 (Table 3, Figure 5). We next combined 15 laboratory clinical features (listed in the methods section) along with the cytokines and reanalyzed the data. This resulted in a new subset (Table 3) and achieved sensitivities and specificities >80% with an AUC of 0.92±0.03 (Table 3, Figure 5). While cytokines IFN-ϒ, IL-1b, IL-8 and IL-17 were common with subset A, combining them with lymphocyte, platelet counts and viral titers improved the predictive performance of subset-B compared to subset-A (Table 3). The addition of two more features- CT and NT- to the dataset resulted in a new subset that retained cytokines- IFN-ϒ and IL-1b, IL-8, and blood lymphocyte count, but also had additional set of cytokines (Table 3) along with CT. However, overall predictive performance of subset group-C was poorer likely due to reduction of population size (n = 54 as compared with n = 62).

**Table 3:** Identification of serum molecules for early classification of DHF.

Subset group	Type of features	Specific features^4^	Sensitivity %*	Specificity %*	AUC*
A	23 Cytokines^1^	IFN-ϒ, IL-1b, IL-17, IL-8,	78(12)	76(8)	0.86(0.05)
		IL-9, Eotaxin, FGF-basic,			
					
B	23 Cytokines^1^	IFN-ϒ, IL-1b, IL-17, IL-8,	80(11)	86(7)	0.92(0.03)
	19 Clinical features^1^	G-CSF, LYMPH, PLT,			
		Viraemia			
C	23 Cytokine^2^	IFN-ϒ, IL-1b, IL-8, IL-9,	71(18)	85(7)	0.88(0.05)
	19 Clinical Features^2^	IL-1ra, G-CSF, Eotaxin,			
	2 Protein Adducts^2^	CT, LYMPH, Viraemia			
D	23 Cytokines^3^	IFN-ϒ, IL-17, FGF-basic,	83(14)	75(13)	0.90(0.06)
	19 Clinical features^3^	RANTES, IP-10, CT,			
	2 protein adducts^3^	LYMPH, SAA, HPT			
	5 Serum proteins^3^				

1 n = 62 (44 DF; 18 DHF);

2 n = 54 (44 DF; 10 DHF);

3 n = 34 (24 DF; 10 DHF);

4 All measurements are from febrile stage (visit-1); Mean (SD) values shown; AUC-area under curve from receiver operator characteristics analysis. DHF - dengue hemorrhagic fever. Please see methods section for details on feature selection.

Finally, we expanded the dataset to include all measured features (i.e. 23 cytokines, 5 serum proteins, 2 protein adducts and 15 clinical features). The number of patients in this analysis was much lower (n = 34) than the previous analysis (n = 62 and n = 54) due to further exclusion of samples where the data was incomplete due to missing values. The subset from this analysis included a variety of features including serum proteins (SAA and HPT), cytokines (IFN-ϒ, IL-17) and protein adducts (CT) that achieved a sensitivity and specificity of >75% and AUC of 0.90±0.06 (Table 3).

## Discussion

We have performed a comprehensive molecular analysis of serum molecules in a cohort of adults with primary dengue infections with the objective of identifying predictive markers of DHF. Traditionally, biomarkers studies have relied mostly on case versus control studies (reviewed in [6]) with one sample per patient, collected in a 1–10 day period. Some of these studies have reconstructed temporal profiles via data grouping based on fever day [19], [20], [21]. However, variability in sample size within groups (e.g. fever day) and lack of patient follow-up often result in poor statistical performance and inadequate modeling of individual immune responses. Prospective follow-up of patients across disease stages, although most desirable for biomarker development, are scarce. A good example is a 1997 pediatric dengue study in Thailand, where a positive dengue diagnosis was followed by daily blood sampling till one day post defervescence [22], [23]. The EDEN study combines the convenience of asynchronous patient recruitment during the early febrile phase, with patient follow-up, and is designed to specifically model adult dengue infections [7].

A detailed cytokine analysis indicated that DHF patients are characterized by an attenuated serum cytokine response especially during the early febrile phase. In DHF, low levels of IFN-γ during febrile phase correlated with reduced levels of IP-10, indicating that an inability to mount a timely anti-viral response may result in high viremia. In cell culture models, pretreatment with interferons inhibits dengue viral replication [24] although treatment after infection has no effect due possibly to active inhibition of IFN-signaling pathways by dengue viral protein NS4B [25]. Whether higher viral titers reported in DHF patients is a consequence or cause of an impaired interferon response remains to be confirmed. In recent human challenge studies, development of infection correlated with extremely low or undetectable IFN-γ production by PBMCs suggesting a role for sustained IFN-γ production in protection [26]. An attenuated innate response may in turn affect the kinetics of adaptive immune and pro-inflammatory responses, as suggested by the lower levels of Th2 cytokines IL-4 and IL-13, growth factors G-CSF, VEGF and PDGF, observed during the febrile stages in DHF patients in this study.

In contrast our findings, a number of previous studies have reported elevated levels of IFN-γ [19], IL-8 [27], IL-6, TNF-a [28], MIP-1b [19], IL-10 [29], and free VEGF [23], in DHF patients. However these studies differ significantly from the present study in the types of clinical cohorts evaluated. For example, comparable longitudinal cohort studies reporting higher levels of IL-10 and IL-6 have focused exclusively on pediatric cases [29], [30]. Importantly, primary infections made up less than ten percent of the cohorts in previous studies reflecting the higher incidence of DHF in secondary infections. Hence, different cytokine profiles observed in the present and previous studies are likely related to differences in immune responses to primary and secondary infections. The few studies that have included a subset of primary infections have reported conflicting results, with some reporting higher levels of cytokines in secondary compared to primary infections [31], [32], while others reporting no differences [33]. It is noteworthy that no DHF patients were included in these studies, and therefore it is not possible to compare cytokine profiles specific to DHF. We hypothesize that timely interferon-regulated antiviral responses are critical determinants of outcome in primary infections, whereas inflammatory mediators and regulators of antibody-dependent enhancement, including IL-6, IL-8, and IL-10 may dominate in secondary infections. Ethnic background of patients can also affect the type of cytokine responses to dengue infections [34], and may contribute to cytokine profiles described here.

In an attempt to identify serum protein markers of DHF, several groups have reported proteomic analysis of dengue patient sera [35], [36], [37]; using a variety of methods and clinical cohorts. We used a highly sensitive isobaric-tag method of quantitation that allowed us the compare the proteomic changes across different stages of infection. We focused on the most prominent functional protein group identified (i.e. acute phase reactants), and observed elevated levels of CRP, SAA, HPT, A2M and FT in individual patient samples. Maximum elevation of CRP and SAA in the early febrile phase was consistent with elevated production of IL-6, a hepatic inflammatory cytokine. Interestingly, acute phase reactants PCT, FB and SAP were not altered, and this may be related to liver dysfunction observed in dengue patients [38]. With the exception of SAA, there were no significant differences between DF and DHF groups suggesting that acute phase response is not a dominant mechanism of pathology in primary infections. Understanding the specific functional role of other proteins shortlisted in our proteomics study will require detailed validation.

Nitric oxide (NO) production by phagocytes is an important inflammatory response to pathogens and although increased levels of both inducible NO synthase (iNOS) and NO levels have been reported in dengue patients [28], [39], their role in dengue viral clearance is unknown. Protein adducts CT and NT formed from NO-mediated reactions are sensitive surrogate measures of neutrophil and macrophage activities during inflammation [18], [40]. We observed elevated levels of CT in dengue patient sera compared to healthy controls, which continued to rise from early febrile to defervescence stage indicating robust and sustained neutrophil activity. Interaction of activated neutrophils with the endothelium has been known to modulate vascular permeability [41], [42]. Whether elevated levels of CT can serve as an early indicator of plasma leakage remains to be tested. In contrast to CT, the transient nature of NT accumulation suggests that macrophage activity is limited to the acute phase of the infection, possibly linked to viral titers.

The comprehensive database of 47 blood parameters from dengue patients described in this study provides a unique opportunity to statistically query this dataset to identify -1) most significant molecules and 2) their relative importance in distinguishing DHF from DF during the early febrile stage. In the final analysis, a subset of 9 features was identified that included 5 cytokines, chlorotyrosine, blood lymphocyte count, and two serum proteins. Overall, cytokines involved in attenuated antiviral response; up regulation of acute phase proteins, and elevated neutrophil activity; together appear to be early signatures of DHF resulting from primary infections. The precise role of other cytokines IL-17, FGF-basic, and RANTES that were included in the predictive subset, in DHF pathogenesis is currently unclear and does not rule out the involvement of other cytokines in regulation of immune mechanisms in DHF patients.

Previously, a variety of statistical methods including classification and regression tree (CART) analyses [43], [44], as well as decision tree algorithms [45] have been used to identify clinical markers that achieved high sensitivity but poor specificity in classification of DHF. These clinical parameters, however, require daily monitoring. Identifying and measuring the molecules that are directly involved in pathogenesis could improve our predictive capabilities. Recently, Brasier et al used a logistic regression approach to report a 3 component biomarker panel consisting of platelet count, lymphocyte count and IL-10 that, classified DF from DHF patients with an accuracy of >85% during the first week following onset of fever [21]. In a second study Brasier et al used a multivariate adaptive regression splines (MARS) method to evaluate cytokines and plasma proteome from a cohort of secondary dengue infections and reported a panel consisting of IL-10 and seven serum proteins that achieved 100% sensitivity and specificity in prediction of DHF in the first week of fever onset [37]. However, these two studies applied a broad window of measurement, which may not capture the dynamic processes of DHF pathogenesis. It also raised the possibility that biomarkers of DHF in secondary infections may be qualitatively and quantitatively different from primary infections. Determining which of these biomarkers reflect differences in primary versus secondary infections and which inform on DHF development, whether in primary or secondary infections, will be critical for the development of robust biomarkers to stratify dengue patients for medical care.

In conclusion, this study describes a comprehensive and systematic molecular analysis of serum samples from a cohort of patients with primary dengue infection. The analytical approach and statistical workflow we have outlined forms a robust platform for both future discovery and validation of biomarkers for prediction of severe dengue disease.

## Supporting Information

Figure S1 **Serum cytokine profile in dengue patients during early febrile, defervescence and convalescent stages of infection.** 27 cytokines measured in sera from 62 dengue patients (44 DF+18 DHF) and 50 asymptomatic healthy controls. Each graph shows data for an individual cytokine plotted as mean values with standard deviation shown in error bars (upper only). Statistical confidence (p<0.05) was analyzed by ANOVA kruskall-wallis test, Dengue vs. healthy control (*p<0.05, **p<0.01, ***p<0.001, NS- not significant).
(TIF)

Figure S2 **Cytokine profiles in dengue patients grouped by fever day.** Samples from the study population were grouped based on the day post onset of fever on which the samples were collected for both DF and DHF groups. A. The number of samples per group for the first seven days and total number of samples for the period 15–30 days post onset of fever are plotted. The days are further annotated to indicate the febrile (visit-1), defervescence (visit-2) and convalescence (visit-3) phases. The levels of select cytokines were also evaluated within these groups and included IFN-ϒ (B), IL-4 (C), IL-1b (D), IL-17 (E) and IL-6 (F). The ANOVA-Kruskal-Wallis test was used to determine confidence levels. DF vs DHF: (* p<0.05, NS- not significant (p>0.1)).
(TIF)

Figure S3 **Community feature selection strategy for comprehensive evaluation of statistical performance of multiple algorithms.** A novel approach was developed to identify predictive biomarkers for dengue disease. This approach involved processing of data through a variety of feature selection methods, each of which generate a shortlist of feature-subsets with varying predictive performance. A ‘subset evaluation’ strategy selects the best subset based on ‘average weighted cost’ following which, hypothesis testing and significance criteria are used to select the ‘best method’.
(TIF)

Table S1 **Serum cytokine levels at various stages of dengue disease in DF and DHF patients.**
(DOCX)

Table S2 **Serum cytokine kinetics in DF and DHF patients compared with healthy individuals.**
(DOCX)

Checklist S1 **Strobe checklist.**
(DOCX)
