# Supplementary figures and images for: Serum Proteome and Cytokine Analysis in a Longitudinal Cohort of Adults with Primary Dengue Infection Reveals Predictive Markers of DHF
Source: PLoS Negl Trop Dis. 2012 Nov 29;6(11):e1887. doi: 10.1371/journal.pntd.0001887 (PMC3510095; doi:10.1371/journal.pntd.0001887)

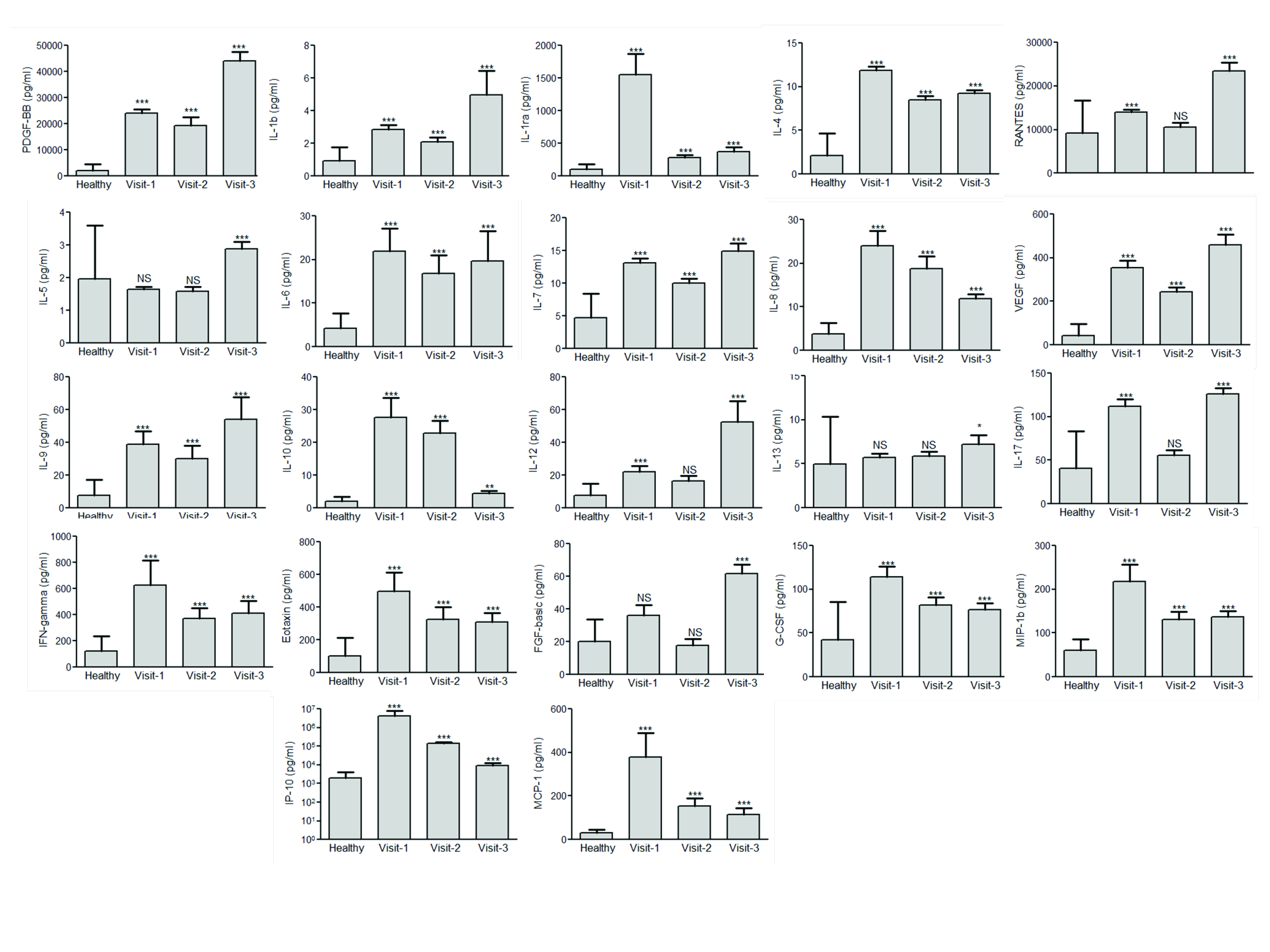

Supplement: Figure S1 — Serum cytokine profile in dengue patients during early febrile, defervescence and convalescent stages of infection. 27 cytokines measured in sera from 62 dengue patients (44 DF+18 DHF) and 50 asymptomatic healthy controls. Each graph shows data for an individual cytokine plotted as mean values with standard deviation shown in error bars (upper only). Statistical confidence (p<0.05) was analyzed by ANOVA kruskall-wallis test, Dengue vs. healthy control (*p<0.05, **p<0.01, ***p<0.001, NS- not significant). (TIF) [file pntd.0001887.s001.tif]

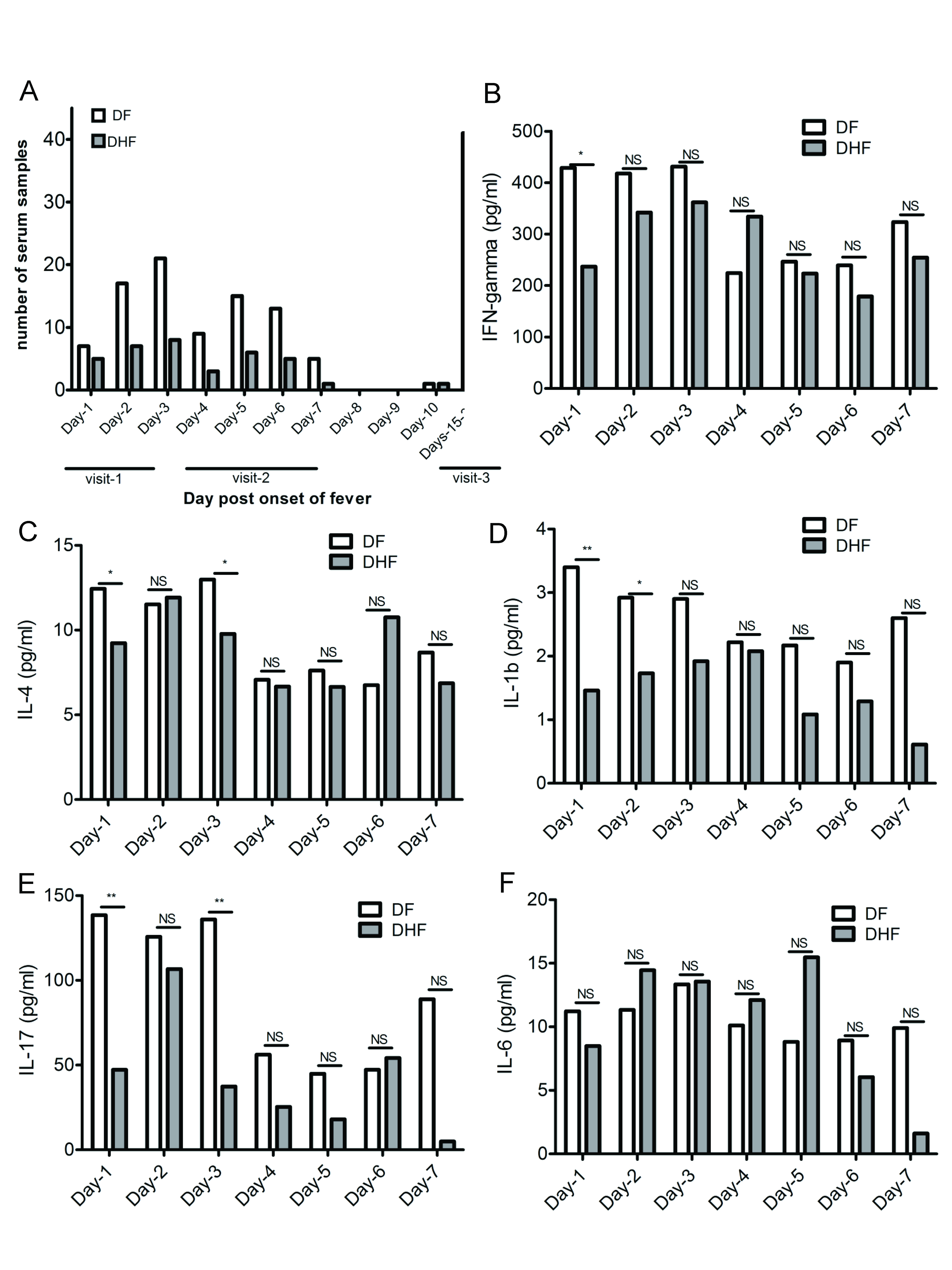

Supplement: Figure S2 — Cytokine profiles in dengue patients grouped by fever day. Samples from the study population were grouped based on the day post onset of fever on which the samples were collected for both DF and DHF groups. A. The number of samples per group for the first seven days and total number of samples for the period 15–30 days post onset of fever are plotted. The days are further annotated to indicate the febrile (visit-1), defervescence (visit-2) and convalescence (visit-3) phases. The levels of select cytokines were also evaluated within these groups and included IFN-ϒ (B), IL-4 (C), IL-1b (D), IL-17 (E) and IL-6 (F). The ANOVA-Kruskal-Wallis test was used to determine confidence levels. DF vs DHF: (* p<0.05, NS- not significant (p>0.1)). (TIF) [file pntd.0001887.s002.tif]

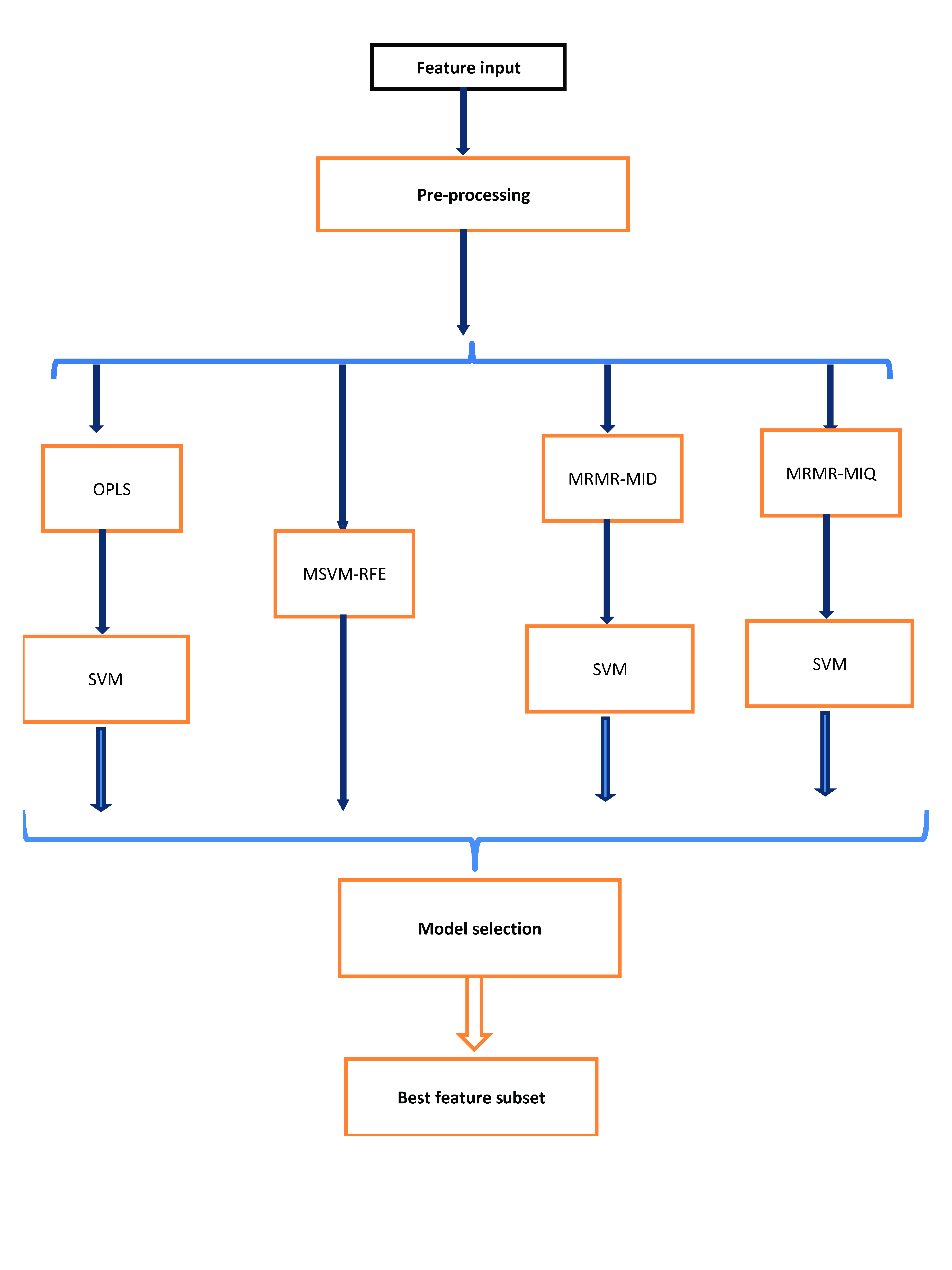

Supplement: Figure S3 — Community feature selection strategy for comprehensive evaluation of statistical performance of multiple algorithms. A novel approach was developed to identify predictive biomarkers for dengue disease. This approach involved processing of data through a variety of feature selection methods, each of which generate a shortlist of feature-subsets with varying predictive performance. A ‘subset evaluation’ strategy selects the best subset based on ‘average weighted cost’ following which, hypothesis testing and significance criteria are used to select the ‘best method’. (TIF) [file pntd.0001887.s003.tif]
