# Supplementary material for: Serum Proteome and Cytokine Analysis in a Longitudinal Cohort of Adults with Primary Dengue Infection Reveals Predictive Markers of DHF
Source: PLoS Negl Trop Dis. 2012 Nov 29;6(11):e1887. doi: 10.1371/journal.pntd.0001887 (PMC3510095; doi:10.1371/journal.pntd.0001887)
Supplement: Table S1 — Serum cytokine levels at various stages of dengue disease in DF and DHF patients. (DOCX) [file pntd.0001887.s004.docx]

**Supporting Table S1: Serum cytokine levels at various stages of dengue disease in DF and DHF patients**

| **CYTOKINE** | **DF (n=44) *** | | | **DHF (n=18) *** | | |
| --- | --- | --- | --- | --- | --- | --- |
|  | **Early Febrile** | **Defervescence** | **Convalescence** | **Early Febrile** | **Defervescence** | **Convalescence** |
| PDGF-BB | 26072.9±8905.8 | 12998.0±7464.3 | 43483.3±20176.2 | 19277±14087.3 | 34311.8±43089 | 45541±3276.1 |
| IL-1b | 3.3±2.2 | 2.3±2.1 | 5.9±13.8 | 1.7±1.0 | 1.4±0.7 | 2.4±1.2 |
| IL-1ra | 1310.1±1393.5 | 287.9±4.9372.5 | 248.1±384.0 | 2137.5±4170.1.0 | 271.7±206.8 | 696.4±798.8 |
| IL-4 | 12.6±2.3 | 8.3±3.8 | 8.8±2.6 | 9.0±4.3 | 8.8 ±4.5 | 10.3±3.2 |
| IL-5 | 1.7±0.8 | 1.5±1.0 | 3.1±1.7 | 1.3±0.5 | 1.6±1.0 | 2.1±1.2 |
| IL-6 | 24.0±47.0 | 18.0±37.1 | 22.4±64.0 | 16.6±24.1 | 14.0±16.8 | 12.3±4.8 |
| IL-7 | 14.0±5.5 | 9.2±4.8 | 13.9±7.7 | 10.7±5.8 | 11.8±7.2 | 17.4±13.1 |
| IL-8 | 21.1±10.1 | 15.8±10.7 | 10.9±7.6 | 31.2±46.8 | 26.0±36.4 | 14.2±7.6 |
| IL-9 | 39.0±66.5 | 21.9±41.6 | 53.6±117.0 | 38.2±55.4 | 49.5±100.0 | 55.6±67.3 |
| IL-10 | 32.5±54.0 | 20.5±26.2 | 3.1±3.0 | 15.5±17.7 | 28.6±38.4 | 7.6±9.2 |
| IL-12 | 23.3±31.3 | 13.1±24.4 | 56.3±117 | 19.0±19.1 | 24.7±21.1 | 41.8±40.0 |
| IL-13 | 6.1±4.0 | 5.7±4.7 | 7.6±9.2 | 4.5±2.2 | 6.0±3.3 | 6.2±5.1 |
| IL-17 | 134.0±54.8 | 60.0±52.4 | 139.1±50.9 | 58.2±51.7 | 43.8±43.0 | 91.9±51.1 |
| Eotaxin | 549.1±1062.7 | 358.7±711.2 | 311.1±446.0 | 369.4±292.4 | 237.8±277.8± | 305.4±337.2 |
| FGF-basic | 46.0±56.1 | 21.3±35.9 | 73.0±40.1 | 11.9±17.5 | 8.0±14.8 | 32.5±35.6 |
| G-CSF | 134.0±103.6 | 94.8±71.7 | 83.3±58.5 | 64.4±50.0 | 51.0±39.2 | 60.0±45.9 |
| IFN-γ | 772.4±1762.7 | 409.2±754.1 | 465.1±857.6 | 263.4±155.6 | 274.0±182.1 | 278.0±112.2 |
| IP-10^1^ | 56.6±278.5 | 1.6±1.0 | 0.04±0.03 | 6.0±8.3 | 0.9±1.9 | 0.2±0.6 |
| MCP-1 | 389.6±929.3 | 167.0±324.4 | 91.7±222.2 | 354.2±703.3 | 116.4±130.5 | 172.2±230.5 |
| MIP-1b | 244.0±362.7 | 139.2±159.5 | 124.8±39.4 | 152.5±109.2 | 112.0±63.4 | 169.7±94.6 |
| RANTES | 15495.3±4750.7 | 10494.2±8378.0 | 24039.0±14573.7 | 10069.5±5315.1 | 10667.0±7013.4 | 21970±16349 |
| VEGF | 381.0±207.2 | 232.4±172.6 | 445.7±267.2 | 292.5±321.1 | 270.5±185.1 | 493.5±595.6 |

* *mean±SD of cytokine levels in patient sera (picogram/ml). ^1^ values (X100000); early febrile stage corresponds to visit-1, Defervescence –visit-2, Convalescence-visit-3. DF dengue fever, DHF dengue hemorrhagic fever.
