# Supplementary material for: Serum Proteome and Cytokine Analysis in a Longitudinal Cohort of Adults with Primary Dengue Infection Reveals Predictive Markers of DHF
Source: PLoS Negl Trop Dis. 2012 Nov 29;6(11):e1887. doi: 10.1371/journal.pntd.0001887 (PMC3510095; doi:10.1371/journal.pntd.0001887)
Supplement: Table S2 — Serum cytokine kinetics in DF and DHF patients compared with healthy individuals. (DOCX) [file pntd.0001887.s005.docx]

**Supporting Table S2: Serum cytokine kinetics at various stages of dengue disease in DF and DHF patients compared with cytokines in an independent healthy plasma cohort**

| **CYTOKINE** | **Fold change DF (n=44) *** | | | **Fold change DHF (n=18) *** | | |
| --- | --- | --- | --- | --- | --- | --- |
|  | **Early Febrile** | **Defervescence** | **Convalescent** | **Early febrile** | **Defervescence** | **Convalescent** |
| PDGF-BB | 35.8±12.2 | 17.8±10.2 | 59.8±27.7 | 26.5±19.3 | 47.2±59.2 | 62.6±45.4 |
| IL-1b | 6.6±4.5 | 4.7±4.3 | 11.8±27.6 | 3.4±1.8 | 2.8±1.4 | 4.8±2.5 |
| IL-1ra | 17.3±18.4 | 3.8±4.9 | 3.2±5.0 | 28.2±55.0 | 3.5±2.7 | 9.1±10.5 |
| IL-4 | 13.7±2.7 | 8.8±4.0 | 9.3±2.7 | 9.6±4.6 | 9.4±4.8 | 10.9±3.5 |
| IL-5 | 1.2±0.5 | 1.1±0.7 | 2.2±1.2 | 0.9±0.4 | 1.2±0.7 | 1.5±0.8 |
| IL-6 | 7.0±13.8 | 5.3±10.9 | 6.6±18.8 | 4.9±7.1 | 4.1±4.9 | 3.6±1.4 |
| IL-7 | 3.0±1.1 | 1.9±1.0 | 3.0±1.6 | 2.3±1.2 | 2.6±1.5 | 3.8±2.8 |
| IL-8 | 6.9±3.3 | 5.1±3.4 | 3.5±2.5 | 10.2±15.3 | 8.5±11.9 | 4.7±2.5 |
| IL-9 | 13.9±23.8 | 7.7±14.9 | 19.2±42.2 | 13.7±19.8 | 17.7±20.6 | 20.0±24.1 |
| IL-10 | 19.4±32.3 | 12.3±15.7 | 1.9±1.8 | 9.3±10.6 | 17.1±20.2 | 4.6±5.5 |
| IL-12 | 3.8±5.1 | 2.1±4.0 | 9.2±19.2 | 3.1±3.1 | 4.0±3.4 | 6.8±6.5 |
| IL-13 | 1.6±1.0 | 1.5±1.2 | 2.0±2.4 | 1.2±0.6 | 1.6±0.8 | 1.7±1.4 |
| IL-17 | 5.4±2.2 | 2.4±2.1 | 5.7±2.0 | 2.3±2.1 | 1.8±1.8 | 3.7±2.1 |
| Eotaxin | 7.0±13.6 | 4.6±9.1 | 3.9±5.7 | 4.7±3.7 | 3.0±3.5 | 4.0±4.3 |
| FGF-basic | 2.2±2.7 | 1.0±1.7 | 3.5±1.9 | 0.5±0.8 | 0.38±0.7 | 1.5±1.7 |
| G-CSF | 5.6±4.3 | 3.9±3.0 | 3.5±2.4 | 2.6±2.0 | 2.1±1.6 | 2.5±1.9 |
| IFN-γ | 8.5±19.3 | 4.5±8.3 | 5.1±9.5 | 2.9±1.7 | 3.0±2.0 | 3.0±1.2 |
| IP-10 | 384±401 | 149±76 | 4.9±2.4 | 459±638 | 72.5±146 | 2.1±1.3 |
| MCP-1 | 14.3±34 | 6.1±11.9 | 3.3±8.1 | 13.0±25.8 | 4.2±4.8 | 6.3±8.4 |
| MIP-1b | 4.5±6.7 | 2.5±2.9 | 2.3±1.8 | 2.8±2.0 | 2.0±1.1 | 3.1±1.7 |
| RANTES | 2.1±0.6 | 1.4±1.1 | 3.3±2.0 | 1.3±0.7 | 1.4±0.9 | 3.0±2.2 |
| VEGF | 23.9±13.0 | 14.6±10.8 | 28.0±16.8 | 18.4±20.2 | 17.0±11.6 | 31.0±37.4 |

* Fold change value = mean of (cytokine levels in patient samples/ healthy controls, median of n=50).

mean (SD) values are shown. DF dengue fever; DHF dengue hemorrhagic fever. Early febrile stage corresponds to

visit-1, Defervescence –visit-2, Convalescence-visit-3.
