# Supplementary material for: Serum Proteome and Cytokine Analysis in a Longitudinal Cohort of Adults with Primary Dengue Infection Reveals Predictive Markers of DHF
Source: PLoS Negl Trop Dis. 2012 Nov 29;6(11):e1887. doi: 10.1371/journal.pntd.0001887 (PMC3510095; doi:10.1371/journal.pntd.0001887)
Supplement: Checklist S1 — Strobe checklist. (DOCX) [file pntd.0001887.s006.docx]

STROBE Statement—Checklist of items that should be included in reports of ***cohort studies***

|  | Item No | Recommendation |
| --- | --- | --- |
| **Title and abstract** | 1 | (*a*) √ See Title |
|  |  | (*b*) √ See Abstract |
| Introduction | | |
| Background/rationale | 2 | √ See Abstract ‘Background’ and Introduction. |
| Objectives | 3 | √ See Introduction |
| Methods | | |
| Study design | 4 | √ See Methods (Patient recruitment) |
| Setting | 5 | √ See Methods (Patient recruitment) |
| Participants | 6 | (*a*) √ See Methods. (Patient recruitment) |
|  |  | (*b*) Not relevant |
| Variables | 7 | √ See Methods section (Patient recruitment and clinical evaluation described defined the disease outcome). Other variables in this study were measured de novo from patient samples. |
| Data sources/ measurement | 8* | √ See Methods section (Cytokine, Proteome measurement, Chloro and Nitro tyrosine measurement, Clinical evaluation are described in separate sections within Methods). All patient groups were treated identically for measurement of variables. |
| Bias | 9 | √ See Methods section (Statistical Analysis), where any bias in dengue serotype distribution, patient age between groups etc., was tested. |
| Study size | 10 | √ See Methods section (Patient recruitment where rationale for study size is described)) |
| Quantitative variables | 11 | √ See Methods section (Cytokine, proteome and chloro and nitrotyrosine measurements; clinical evaluation and variables) |
| Statistical methods | 12 | (*a*) √ See Methods section (Statistical analysis) |
|  |  | (*b*) √ See Methods section (Statistical analysis) |
|  |  | (*c*) √ See Methods section (Statistical analysis) |
|  |  | (*d*) Not relevant |
|  |  | (*e*) √ See Methods section (Statistical analysis) |
| Results | | |
| Participants | 13* | (a) √ See Results section (Parts-I-V) ; Table-1; at each stage number of individuals in the study are described. |
|  |  | (b) Not relevant |
|  |  | (c) Not relevant |
| Descriptive data | 14* | (a) Table-1 |
|  |  | (b) √ See Methods section (Cytokine measurement); Results section (Part-I) |
|  |  | (c) √ Table-I |
| Outcome data | 15* | Not relevant |
| Main results | 16 | (*a*) √ See Results section (Parts-I-IV); Tables 1-3, and figures-1 to 5. Details also in Method section (Statistical analysis) |
|  |  | (*b*) Not relevant |
|  |  | (*c*) Not relevant |
| Other analyses | 17 | √ Results (Part-V), Also details in Methods Section (statistical analysis) |
| Discussion | | |
| Key results | 18 | √ See discussion section Paragraphs-2, 4, 5 and 6 |
| Limitations | 19 | √ See discussion section Paragraphs-3, 4 and 7 |
| Interpretation | 20 | √ See discussion section |
| Generalisability | 21 | √ See discussion section Paragraph7 |
| Other information | | |
| Funding | 22 | Funding source-"This research was supported by the National Research Foundation Singapore through the Singapore MIT Alliance for Research and Technology's <ID IRG> research programme." |

*Give information separately for exposed and unexposed groups.

**Note:** An Explanation and Elaboration article discusses each checklist item and gives methodological background and published examples of transparent reporting. The STROBE checklist is best used in conjunction with this article (freely available on the Web sites of PLoS Medicine at http://www.plosmedicine.org/, Annals of Internal Medicine at http://www.annals.org/, and Epidemiology at http://www.epidem.com/). Information on the STROBE Initiative is available at http://www.strobe-statement.org.
